# Clinical outcomes of patients with advanced synovial sarcoma or myxoid/round cell liposarcoma treated at major cancer centers in the United States

**DOI:** 10.1002/cam4.3039

**Published:** 2020-05-06

**Authors:** Seth M. Pollack, Neeta Somaiah, Dejka M. Araujo, Mihaela Druta, Brian A. Van Tine, Melissa A. Burgess, Sant P. Chawla, Mahesh Seetharam, Scott H. Okuno, Chet Bohac, Michael Chen, Sergey Yurasov, Steven Attia

**Affiliations:** ^1^ Fred Hutchinson Cancer Research Center Seattle WA USA; ^2^ MD Anderson Cancer Center Houston TX USA; ^3^ Moffitt Cancer Center Tampa FL USA; ^4^ Washington University Alvin J. Siteman Cancer Center St. Louis MO USA; ^5^ University of Pittsburgh Pittsburgh PA USA; ^6^ Sarcoma Oncology Center Santa Monica CA USA; ^7^ Mayo Clinic Phoenix AZ USA; ^8^ Mayo Clinic Rochester MN USA; ^9^ Immune Design South San Francisco CA USA; ^10^ Macrogenics Rockville MD USA; ^11^ Mayo Clinic Jacksonville FL USA; ^12^ Present address: Nuvation Bio San Francisco CA USA

**Keywords:** database, medical records, myxoid, neoplasms, registries, sarcoma, synovial

## Abstract

**Background:**

Outcomes data regarding advanced synovial sarcoma (SS) and myxoid/round cell liposarcoma (MRCL) are limited, consisting primarily of retrospective series and post hoc analyses of clinical trials.

**Methods:**

In this multi‐center retrospective study, data were abstracted from the medical records of 350 patients from nine sarcoma centers throughout the United States and combined into a registry. Patients with advanced/unresectable or metastatic SS (n = 249) or MRCL (n = 101) who received first‐line systemic anticancer therapy and had records of tumor imaging were included. Overall survival (OS), time to next treatment, time to distant metastasis, and progression‐free survival (PFS) were evaluated using the Kaplan‐Meier method and Cox regression.

**Results:**

At start of first‐line systemic anticancer therapy, 92.4% of patients with SS and 91.1% of patients with MRCL had metastatic lesions. However, 74.7% of patients with SS and 72.3% of patients with MRCL had ≥2 lines of systemic therapy. Median OS and median PFS from first‐line therapy for SS was 24.7 months (95% CI, 20.9‐29.4) and 7.5 months, respectively (95% CI, 6.4‐8.4). Median OS and median PFS from start of first‐line therapy for MRCL was 29.9 months (95% CI, 27‐44.6) and 8.9 months (95% CI 4.5‐12.0).

**Conclusions:**

To the best of our knowledge, this is the largest retrospective study of patients with SS and MRCL. It provides an analysis of real‐world clinical outcomes among patients treated at major sarcoma cancer centers and could inform treatment decisions and design of clinical trials. In general, the survival outcomes for this selected population appear more favorable than in published literature.

## Graphical abstract

This comprehensive analysis of real‐world outcomes in patients with advanced synovial sarcoma and myxoid round cell liposarcoma provides greater insight into clinical outcomes than previously published research. These data have the potential to inform prognosis and treatment decisions, as well as provide guidance in the development of future clinical trials.

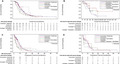

## 1 INTRODUCTION

Synovial sarcoma (SS) and myxoid/round cell liposarcoma (MRCL) are the two most common translocation‐driven soft tissue sarcoma subtypes and share a number of important clinical and biologic features.^1^ They have a similar annual incidence rate of 800‐1000 cases per year.^2^, ^3^ Both SS and MRCL occur most commonly in young adults and are often initially sensitive to chemotherapy. However, both diseases can be aggressive and have dismal outcomes in the refractory metastatic settings.^4^, ^5^, ^6^, ^7^ Overall, the median overall survival (OS) for soft tissue sarcoma is 4‐18.5 months for patients treated with chemotherapy.^8^ In an analysis of seven randomized trials on first‐line chemotherapy by the European Organisation for Research and Treatment of Cancer, the median survival was 51 weeks for advanced soft tissue sarcoma and the overall response rate was 26%.^9^ For newly diagnosed patients with advanced disease, a median survival of 22 months and a response rate of 58.6% to doxorubicin plus ifosfamide were reported.^10^ In a recent study comparing doxorubicin plus evofosfamide to doxorubicin that enrolled 640 patients, the OS was 18.4 months vs 19 months, respectively, and progression‐free survival (PFS) was 6.3 months vs 6 months.^11^ In a separate analysis of 15 randomized clinical trials, the response to chemotherapy was 27.8% for patients with SS vs 18.8% for all patients, with an OS of 15.0 months for SS vs 11.7 months for all subjects and PFS of 6.3 months vs 3.7 months, respectively.^5^ For SS, the time to next treatment after progression on first‐line therapy was 8.3 months, with an OS at time of starting second‐line treatment of 18.6%.^4^

The natural history of MRCL is less well defined in the literature. In a trial of trabectedin for metastatic liposarcoma or leiomyosarcoma, the PFS for patients treated with trabectedin was 4.2 months vs 1.5 months for dacarbazine and the OS was 12.4 months vs 12.9 months. In this study, only 11% of enrolled subjects were of the MRCL histologic subtype.^12^ In another Phase 3 trial, the OS was 13.5 months for eribulin vs 11.5 months for dacarbazine, and PFS was 2.6 months for both the eribulin and dacarbazine arms.^13^ However, only 12% of patients had MRCL.

Recently, the biology of these two cancers has raised interest in the development of tumor‐specific research in SS and MRCL. Emerging data has revealed key insights into the biology of the translocations that drive these tumors, suggesting that molecularly targeted approaches and epigenetic modifications could be promising treatment strategies.^14^, ^15^, ^16^ Immunotherapy also may be a potential treatment for these cancers, since they both have high rates of the target protein NY‐ESO‐1.^17^

Synovial sarcoma and MRCL are unique among malignancies in their confirmed high incidence of NY‐ESO‐1 homogeneous expression (>80%) patterns. Both SS and MRCL also appear to have immune microenvironments with few infiltrating T cells and low levels of antigen presentation proteins.^18^, ^19^ As a result of these findings, there is significant interest in evaluating the expression of cytokines and other immunomodulators in these cancers.

A review of the literature regarding SS and MRCL clinical outcomes identified only retrospective series and post hoc subgroup analyses from randomized clinical trials.^3^, ^4^, ^5^, ^6^, ^7^, ^8^, ^10^, ^20^, ^21^ In SS and MRCL, there is an unmet need to better understand clinical outcomes to inform clinical management of these sarcomas and design prospective clinical studies.^3^, ^7^ In clinical trial data sets specific to SS and/or MRCL, the quality of data is limited by the heterogeneity of patients, sparse staging information, and varying length of survival follow‐up, which limits the generalizability of results.^3^, ^4^, ^5^, ^6^, ^7^, ^8^, ^10^, ^20^, ^21^ Recent studies have reported the need for improved understanding of the tumor biology, pattern of spread, prognostic factors, and treatments that lead to improved outcomes in SS and MRCL.^3^, ^6^, ^7^

The objective of this multi‐center, retrospective registry study was to document the clinical outcomes of patients with advanced SS or MRCL treated with at least 1 line of systemic anticancer therapy using data from real world experience. These data could then inform the design of trials with novel agents in the future.

## 2 STUDY METHODS

### 2.1 Patient identification and data abstraction

Data were abstracted from medical records of 350 patients diagnosed with either SS or MRCL and treated for locally advanced/unresectable or metastatic disease at nine United States cancer centers that specialize in management of soft tissue sarcoma. The information was recorded so that patients could not be identified directly or through identifiers linked to the patients. As such, Exemption 45 Code of Federal Regulations 46.101(b)(4) applies to this study. This study was eligible for HIPAA waiver and expedited Institutional Review Board review. The Institutional Review Boards at the participating study sites approved this study.

Each center identified patients diagnosed with SS or MRCL for eligibility review. Patients diagnosed with advanced SS or MRCL and treated with at least 1 line of systemic anticancer therapy for locally advanced/unresectable or metastatic disease between 1 January 2005 and 31 December 2015 were eligible for inclusion in the study. The diagnosis of SS and MRCL for each patient was required to be validated by a documented signed pathology report detailing the diagnosis. Surgical pathology notes and/or radiology reports were used to determine the disease status. Tumors were considered locally advanced or unresectable if the resection margin of the tumor had evidence of positive tumor in the surgical margin or if the primary tumor was deemed inoperable per a source‐verified clinic note documenting that the tumor was not operable with no evidence of distant metastasis. Stage IV or metastatic disease was determined using evidence from either a radiology report that documented at least one or more distant metastatic lesions or through clinic notes that stated that the tumor was metastatic prior to the start of systemic anti‐cancer therapy. Patients were excluded from the study if they had a history of another cancer within 2 years prior to their SS or MRCL diagnosis, except excised in situ carcinoma or non‐melanoma cutaneous malignancies. Patients without clinical records of tumor imaging or systemic anticancer therapy administration were excluded.

All data on patients who met eligibility criteria were analyzed for clinical outcomes after each line of systemic anticancer therapy. The primary endpoint was OS, which was defined as the time from date of first administration of the first line of systemic anticancer therapy until death due to any cause. Patients without death were censored at last known alive date. PFS was defined as the time from date of first administration of each line of systemic anticancer therapy to progression or death. Patients without progression or death were censored at the start date of the next line of systemic anticancer therapy, or if there was no additional systemic anticancer therapy administered, the patients were censored at the end date of the current line of systemic anticancer therapy.

Secondary endpoints were time to next treatment (TTNT), time to distant metastasis (TTDM), and PFS. TTNT was defined as the time from date of first administration of each line of systemic anticancer therapy to the next subsequent systemic anticancer therapy, and patients who did not have further therapy were censored at last known alive date. TTDM was defined as the time from first administration of systemic anticancer therapy to a new distant organ with evidence of a new lesion. Patients without distant metastasis were censored at the start date of the next line of systemic anticancer therapy or censored at the end date of the current line of systemic anticancer therapy if there were no additional lines of systemic anticancer therapy.

Exploratory endpoints were prognostic factors, clinical benefit rate (complete response + partial response + stable disease), pattern of tumor recurrence, and use of supportive care.

The tumor response for each line of systemic anti‐cancer therapy was determined using the treating oncologist's recorded assessment by clinical exam and/or radiographic imaging, such as computed tomography and magnetic resonance imaging. The following information and assessments for each patient were included in the registry data: demographics, date of birth, sex, race, performance status, tumor histology, medical history, tumor history, and clinical visits.

The data analysis was conducted from March 2017 to December 2017 using SAS 9.4. Kaplan‐Meier methodology was used to evaluate the data for clinical outcomes (OS, PFS, TTNT, TTDM). Cox regression was used to estimate hazard ratios. Forest plots were generated to evaluate the impact of baseline characteristics on clinical outcomes.

## 3 RESULTS

### 3.1 Patient demographics

There were 249 patients with SS and 101 patients with MRCL who received first‐line systemic anticancer therapy included in the study. The median age at diagnosis for SS and MRCL was 40 years (range 19, 86) and 50 years (range 27, 82), respectively, and percent male for SS and MRCL was 58.2% and 67.3%, respectively.

The stage at diagnosis for patients with SS was 2.0% stage I, 14.5% stage II, 39.0% stage III, and 24.5% stage IV. Among patients with SS, 85.1% had evidence of metastatic disease at start of first‐line systemic anticancer therapy, and the rest had locally advanced/unresectable disease. The stage at diagnosis for patients with MRCL was 2.0% stage I, 21.8% stage II, 29.7% stage III, and 17.8% stage IV. Among patients with MRCL, 91.1% had evidence of metastatic disease at start of first‐line systemic anticancer therapy, and the rest had locally advanced/unresectable disease.

**FIGURE 1:**
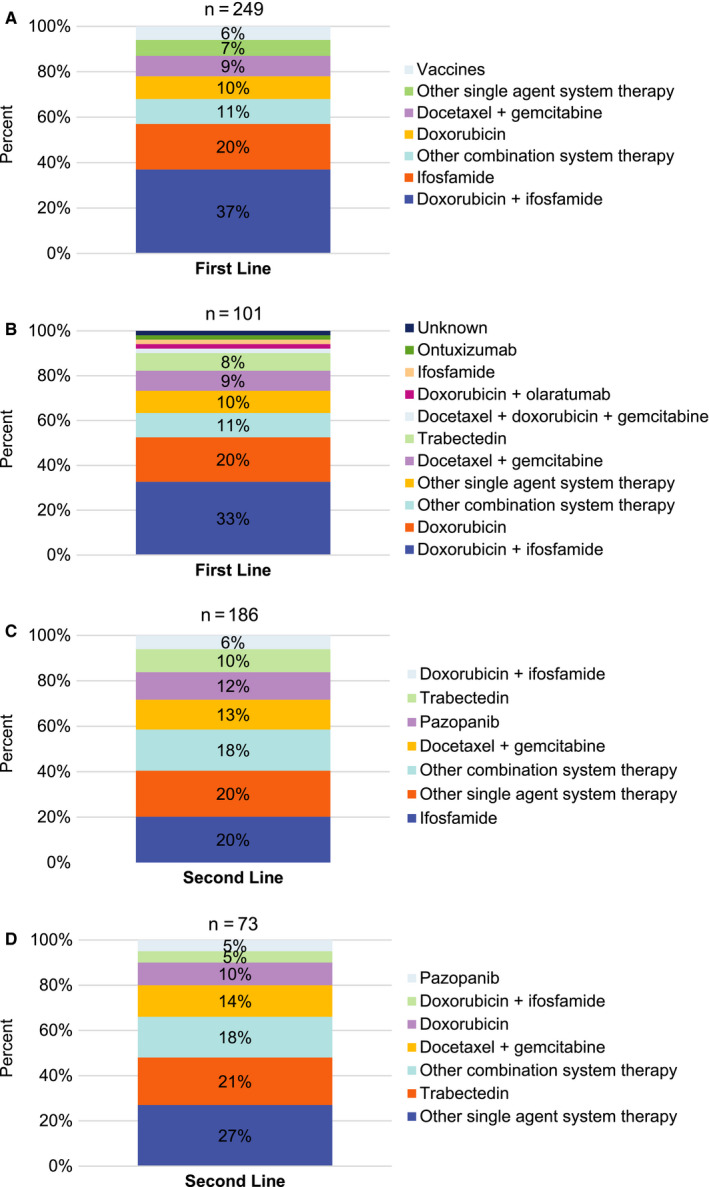
Anticancer therapy (top 5 therapies vs. other therapy) in first‐ and second line. A, Synovial sarcoma: anticancer therapy (top 5 vs other) in first line. In first line, the single agents included in "Other single agent systemic therapy" category are atezolizumab, cisplatin, gemcitabine, interferon gamma, ontuxizumab, other hormone antagonists and related agents, pazopanib, and trabectedin. B, Myxoid/round cell liposarcoma: anticancer therapy (top 5 vs other) in first line. In first line, the single agents included in "Other single agent systemic therapy" category are dacarbazine, gemcitabine, monoclonal antibodies, other vaccines, oxaliplatin, paclitaxel, pazopanib, ridaforolimus, temozolomide, and vaccines. C, Synovial sarcoma: anticancer therapy (top 5 vs other) in second line. In second line, the single agents included in "Other single agent systemic therapy" category are antilymphocyte immunoglobulin, atezolizumab, dacarbazine, doxorubicin, epirubicin, gemcitabine, immunostimulants, monoclonal antibodies, nivolumab, NY‐ESO‐1, other vaccines, pazopanib hydrochloride, sirolimus, sorafenib, temozolomide, and vaccines. D, Myxoid/round cell liposarcoma: anticancer therapy (top 5 vs other) in second line. In second line, the single agents included in "Other single agent systemic therapy" category are atezolizumab, dacarbazine, dasatinib, gemcitabine, ifosfamide, olaratumab, pembrolizumab, protein kinase inhibitors, selumetinib, sorafenib, sunitinib, and vaccines

The 249 patients with SS all had ≥1 line of systemic anticancer therapy since this was required for study entry, and 74.7% had ≥2 lines of therapy (Figure 1). The most common first‐line and second‐line systemic anticancer therapy for SS was doxorubicin plus ifosfamide and single agent ifosfamide, respectively. Many patients with SS had treatment other than systemic anticancer therapy during any line of therapy, including metastectomy (n = 114 [45.8%]), radiation (n = 52 [20.9%]), radiofrequency ablation (n = 7 [2.8%]), and cryoablation (n = 2 [0.8%]).

The 101 patients with MRCL all had ≥1 line of systemic anticancer therapy required for study entry, and 72.3% had ≥2 lines of therapy (Figure 1). The most common first‐line and second‐line systemic anticancer therapies for patients with MRCL were doxorubicin plus ifosfamide and other single agents, respectively. The use of treatment other than systemic anticancer therapy for the patients with MRCL during any line of therapy included metastectomy (n = 53 [52.5%]) and radiation therapy (n = 26 [25.7%]). No patient with MRCL received radiofrequency ablation or cryotherapy.

### 3.2 Patient outcomes

Overall survival, PFS, and other clinical outcomes (TTNT and TTDM) for patients with SS or MRCL from start of first‐line systemic anticancer therapy are listed in Table 1. The median 12‐month OS rates for patients with SS and MRCL were 84.6% (95% CI, 79.3‐88.6) and 84.6% (95% CI, 75.7‐90.4), respectively, and the median 12‐month PFS rates were 26.3% (95% CI, 20.2‐32.7) for patients with SS and 37.5% (95% CI, 27.4‐47.6) for patients with MRCL.

**TABLE 1:** Survival and other clinical outcomes from start of first‐line systemic anticancer therapy

Disease type	All patients	Locally advanced, unresectable	Metastatic
	Median months (95% CI)		
Synovial sarcoma	N = 249	N = 37	N = 212
Overall survival	24.7 (20.9, 29.4)	33.8 (21.3, 49.1)	23.4 (19.9, 28.0)
Progression‐free survival	7.5 (6.4, 8.4)	8.6 (6.4, 12.4)	7.5 (5.7, 8.3)
Time to next treatment	9.2 (7.9, 10.2)	10.1 (6.9, 22.0)	9.2 (7.9, 10.2)
Time to distant metastasis	21.8 (14.6, 32.6)	14.6 (10.3, 29.3)	27.1 (17.3, NR)
Myxoid/round cell liposarcoma	N = 101	N = 9	N = 92
Overall survival	29.9 (27, 44.6)	NR (4.6, NR)	29.2 (26.2, 35.1)
Progression‐free survival	8.9 (4.5, 12.0)	12.5 (1.4, 17.0)	8.0 (4.0, 11.5)
Time to next treatment	12.2 (7.3, 15.2)	27.1 (1.4, 70.8)	11.0 (6.4, 14.5)
Time to distant metastasis	17.0 (12.5, 52.4)	17.0 (6.9, NR)	17.5 (12.0, 52.4)

Abbreviations: CI, confidence interval; NR, not reached.

The median OS for the 165 patients with SS from start of second‐line systemic anticancer therapy was 16.0 months (95% CI, 13.5‐18.9) and the median PFS for these patients was 3.9 months (95% CI, 3.4‐5.1). The median OS for the 73 patients with MRCL from the start of second‐line systemic anticancer therapy was 25.7 months (95% CI, 19.2‐32.1) and the PFS for these patients was 3.5 months (95% CI, 2.7‐5.5).

**FIGURE 2:**
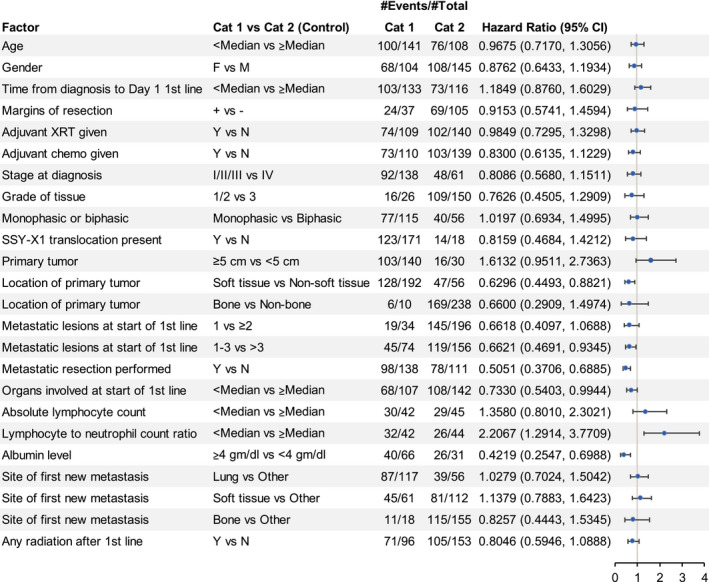
Synovial sarcoma: overall survival and baseline characteristics. Among the synovial sarcoma patients, location of the primary tumor, metastatic lesions at start of first‐line therapy, metastatic resection performed, lymphocyte to neutrophil count, and albumin level appear to be prognostic for overall survival in this exploratory analysis. The #Events indicates the number of patients experiencing the event (ie, death) within each category and the #Total is the total number of patients in each category. Cat1, category 1; Cat2, category 2; Chemo, chemotherapy; CI, confidence interval; F, female; M, male; N, no; XRT, radiotherapy; Y, yes

**FIGURE 3:**
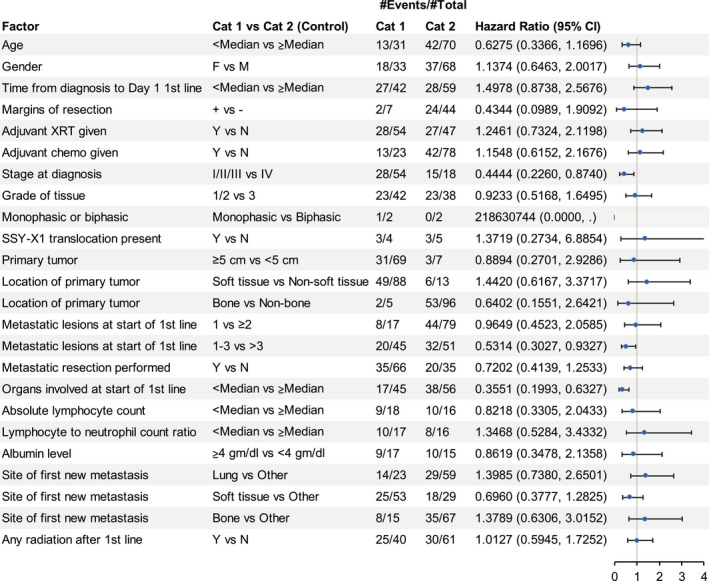
Myxoid/round cell liposarcoma: overall survival and baseline characteristics. For the patients with myxoid/round cell liposarcoma, stage at diagnosis, metastatic resection performed, and organs involved at start of first‐line therapy appear to be prognostic for overall survival in this exploratory analysis. The #Events indicates the number of patients experiencing the event (ie, death) within each category and the #Total is the total number of patients in each category. Cat1, category 1; Cat2, category 2; Chemo, chemotherapy; CI, confidence interval; F, female; M, male; N, no; XRT, radiotherapy; Y, yes

Forest plots of OS outcomes from first‐line systemic anticancer therapy for SS and MRCL patients enrolled in the trial by baseline characteristics and tumor history are presented in Figures 2 and 3.

For patients with SS, the most frequent site of recurrence after first‐ and second‐line systemic anticancer therapy was the lung in 37.3% and 20.5% of patients, respectively. For MRCL, the most frequent sites of recurrence after first‐ and second‐line therapy were soft tissue (54.8%) and lung (19.8%), respectively. The mean number of hospitalizations during first‐line systemic anticancer therapy was 1.4 (standard deviation 1.19) for SS and 1.7 (standard deviation 1.79) for MRCL. The median number of blood transfusions during first‐line systemic anticancer therapy was 1.5 (range: 1, 10) for patients with SS and 1.0 (range: 1, 2) for patients with MRCL.

**FIGURE 4:**
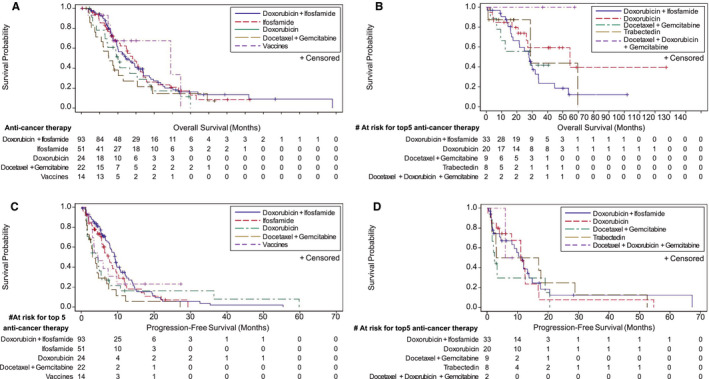
Overall Survival and Progression‐Free Survival by First‐Line Therapy. This figure portrays overall survival and progression‐free survival by the top five first‐line therapies with more than 25 patients. The overall survival and progression‐free survival rates for each subgroup at a given timepoint can be determined by visually tracing a vertical line from the timepoint on the X‐axis to the line for the subgroup; that location of the subgroup line on the Y‐axis indicates the rate. A, Overall survival in patients with synovial sarcoma by first‐line therapy. B, Overall survival in patients with myxoid/round cell liposarcoma by first‐line therapy. C, Progression‐free survival in patients with synovial sarcoma by first‐line therapy. D, Progression‐free survival in patients with myxoid/round cell liposarcoma by first‐line therapy

Figure 4 shows survival outcomes for the top 5 regimens (with at least 25 patients) used for first‐line systemic anticancer therapy among patients with SS or MRCL. Among patients who received doxorubicin plus ifosfamide (the top first‐line therapy), the 12, 24, and 36‐month survival rates for patients with SS were 90.0% (95% CI, 81.7‐94.7), 51.7% (95% CI, 40.5‐61.8), and 32.5% (95% CI, 22.0‐43.3) respectively, and for patients with MRCL, 90.6% (95% CI, 73.6‐96.9), 59.6% (95% CI, 39.8‐74.8), and 23.3% (9.1‐41.2), respectively.

## 4 DISCUSSION

This retrospective study analyzed clinical outcomes of SS and MRCL patients treated with systemic anticancer therapy in routine clinical practice at major sarcoma care medical centers. Nine centers from across the United States were included, and an exploratory analysis of clinical outcomes by center did not demonstrate significant differences for first‐ or second‐line patients with SS or for second‐line patients with MRCL. However, differences due to variation in number of patients included by site were seen for first‐line patients with MRCL in 1 center (n = 2) that had a 54.4‐month median OS and another center (n = 23) with 11.7 months.

The patients included in this study were older than the expected age for SS, which in most studies usually range from age 15 to 30 years old. The estimated median OS from the SS cohort from first‐line treatment (n = 249) was 24.7 months (95% CI, 20.9‐29.4) and the median PFS was 7.5 months (95% CI, 6.4‐8.4). These survival outcomes appear greater than those published from previous retrospective series and clinical trials.^3^, ^4^, ^5^, ^6^, ^7^, ^8^, ^22^, ^23^, ^24^

One of the most recent studies that included a significant cohort of patients with locally advanced or metastatic SS (n = 313) reported a median OS of 15.0 months and a median PFS of 6.3 months.^5^ The proportion of patients with SS in our study who had metastases (85.1%) was comparable to that seen in the study by Vlenterie et al, but patients with metastases in our study had a greater median OS vs the Vlenterie study (23.4 months vs 14.9 months). One of the few recent retrospective studies with only metastatic soft tissue sarcoma patients by Savina et al^25^ showed that the cohort of patients with SS (n = 188) had a reported OS of 19.7 months.

In this study's MRCL cohort, the median OS and median PFS from first‐line treatment were 29.8 months (95% CI, 27.0‐44.6) and 8.9 months (95% CI 4.5‐12.0), respectively. It is difficult to compare the outcomes for the MRCL cohort in this study to other data due to the small number of patients with MRCL in published studies.^12^, ^13^, ^26^, ^27^ One of the most detailed studies on MRCL was a retrospective review of 268 enrolled patients with localized or metastatic disease treated at the University of Texas MD Anderson Cancer Center between 1990 and 2010.^7^ Of these, 61 patients had metastatic disease and their disease‐specific survival was 78% at 1 year and 8.2% at 5 years. None of the patients survived over 9 years.

The survival outcomes in this study, when analyzed by systemic anticancer therapy, appear longer than in previously published data as well. In this study, most patients received systemic anticancer therapy per National Comprehensive Cancer Network guidelines for management of their subtype of soft tissue sarcoma. An important consideration is that there were systemic anticancer therapies not yet approved during the time for which data were abstracted, such as trabectedin for the treatment of liposarcoma, which was not approved until October 2015.

In this study, the median OS for patients with SS in first‐line systemic anticancer therapy on doxorubicin plus ifosfamide (n = 93), single agent ifosfamide (n = 51), and single agent doxorubicin (n = 24) was 24.5, 28.0, and 20.0 months, respectively. For patients with MRCL in first‐line systemic anticancer therapy, the median OS for doxorubicin plus ifosfamide (n = 33), single agent doxorubicin (n = 20), and single agent trabectedin (n = 8) was 27.8, 54.6, and 28.8 months, respectively.

Comparisons to outcomes for similar therapies in the published literature are difficult due to the paucity of studies with more than 50 enrolled patients with SS or MRCL that reported survival outcomes by systemic anticancer therapy. No studies to date with more than 50 enrolled patients with MRCL have reported outcomes with different anticancer therapies.

However, a standard systemic anticancer therapy for advanced soft tissue sarcoma is doxorubicin plus ifosfamide, which had a median OS of 14.3 months (12.5‐16.5) and median PFS of 7.4 months (95% CI, 6.6.‐8.3) in Judson et al (n = 228).^28^ A systematic review of anticancer therapy in SS identified only 1 study with greater than 50 patients with advanced SS who received therapies like those in this study.^6^ In the 2016 Vlenterie study, patients on the top three anticancer regimens—anthracyline‐based treatment (n = 121), doxorubicin plus ifosfamide (n = 112), and ifosfamide alone (n = 42)—had a median OS of 14.85, 14.98, and 15.34 months, respectively.^5^

The use of registries is well validated for understanding clinical outcomes in patients with cancer and rare diseases such as SS and MRCL. Information on clinical outcomes, including remission and death, can be obtained if the patients are followed routinely, either directly or with other registries or administrative databases.^29^ Retrospective registries are also a reliable and valuable complement to randomized clinical trials in determining real world outcomes.^30^

A comparison of clinical outcomes in our study to the published data is also not possible, since the selection of treatment in this study was not determined by predefined eligibility criteria. In addition, this study is limited by variation in real world practice between medical centers and the use of other types of anticancer therapy and locoregional procedures, typically proscribed in a clinical trial. This study found that a significant percent of patients received either metastectomies or addition of radiation or other local therapy in the management of disease burden, which likely improved clinical outcomes.

Another consideration is that the eligibility criteria for this study were designed to ensure inclusion of only pathologically confirmed patients with SS or MRCL, who received at least 1 line of systemic anticancer therapy and were followed until death or the last confirmed survival documented. This allowed for a more complete follow‐up of survival than clinical trials in which patients who have discontinued investigational products are lost to follow‐up or not followed until death. These factors likely contributed to the greater survival outcomes in this study.

The statistics gathered were broad and detailed, including TTNT and TTDM for all patients in the study; these variables were also compared in patients with locally advanced disease vs metastatic disease. This analysis indicated that the time to evidence of a new distant metastatic lesion was shortened for locally advanced/unresectable SS disease compared to patients with SS with metastatic disease. This was most likely due to the metastatic lesion not being discovered in body areas not included on imaging at initiation of first‐line systemic anticancer therapy (median TTDM 14.6 vs 27.1 months for patients with SS with locally advanced and metastatic disease, respectively). Time to distant metastases among patients with MRCL with locally advanced/unresectable disease and metastatic disease was 17 and 17.5 months, respectively.

Other sources of bias and confounding include the variation in clinical practice across the cancer centers. The nine centers in the study did not use the same methods for systematic collection of data in the medical records of patients with SS or MRCL. Nor were the same protocols used for establishing dosing schedules of the systemic anticancer therapy. Treating oncologists varied in their selection of systemic anticancer therapy and locoregional therapy, the length of time to continue treatment, and when to initiate or discontinue treatment after tumor progression. Response Evaluation Criteria in Solid Tumors (RECIST) v1.1 was not used as a measure of response nor were death certificates available, and thus verification was not performed. Death was documented from tumor registry updates and documentation in medical records, and sources were verified.

While just nine centers were involved in the study, all regions in the United States were represented, including the Northwest, West, Midwest, East, South, and Southeast. The variation in locale and the study's reliance on real‐world outcomes makes it more likely that the data are an accurate reflection of oncology practice today.

## 5 CONCLUSION

This is the first large‐scale systematic retrospective study including patients with locally advanced/unresectable or metastatic SS or MRCL. The survival outcomes in this study appear improved over previously published data. Thus, this study provides greater insight into SS and MRCL clinical outcomes than previously published studies and has the potential to inform prognosis and treatment decisions, as well as provide guidance in the development of future therapeutic clinical trials.

## CONFLICT OF INTEREST

Chet Bohac, Michael Chen, and Sergey Yurasov are current or former employees of Immune Design Corp. Seth Pollack has received honoraria from Seattle Genetics, Bayer, Tempus, Daiichi Sankyo, and Blueprint, and grants and research funding from Merck, EMD Sereno, Incyte, Presage, Janssen, Oncosec, and Juno Therapeutics. Mihaela Druta has received consulting fees from Immune Design Corp. Brian Van Tine reports basic science grant funding from Pfizer, Tracon, and Merck; consulting fees from Epizyme, Lilly, CytRx, Janssen, Immune Design, Daiichi Sankyo, Plexxikon, and Adaptimmune; speaking fees from Caris, Janssen, and Lilly; and travel support from Lilly, GSK, and Adaptimmune. Sant Chawla has received honoraria and research funding from Immune Design Corp., Amgen, CytRx Corporation, GlaxoSmithKline, Ignyta, Janssen, Karyopharm Therapeutics, Roche, Sarcoma Alliance for Research through Collaboration, Threshold Pharmaceuticals, and TRACON Pharma. Steven Attia has received research funding from the Desmoid Tumor Research Foundation, AB Science, TRACON Pharma, CytRx Corporation, Bayer, Novartis, Daiichi Sankyo, Lilly, Immune Design, Karyopharm Therapeutics, Epizyme, Blueprint Medicines, Genmab, CBA Pharma, Merck, Philogen, Gradalis, Deciphera, Takeda, Incyte, Springworks, Adaptimmune, Advenchen Laboratories, Bavarian Nordic, BTG, PTC Therapeutics, GlaxoSmithKline, and FORMA Therapeutics. The other authors have no conflicts of interest to disclose.

## AUTHOR CONTRIBUTIONS

Seth Pollack: conceptualization, formal analysis, writing—review and editing. Neeta Somaiah: conceptualization, writing—review and editing. Dejka Araujo: conceptualization, formal analysis, writing—review and editing. Mihaela Druta: conceptualization, formal analysis, writing—review and editing. Brian Van Tine: conceptualization, formal analysis, writing—review and editing. Melissa Burgess: conceptualization, formal analysis, writing—review and editing. Sant Chawla: conceptualization, formal analysis, writing—review and editing. Mahesh Seetharam: conceptualization, formal analysis, writing—review and editing. Scott Okuno: conceptualization, data curation, formal analysis, validation, writing—review and editing. Chet Bohac: conceptualization, data curation, formal analysis, investigation, methodology, project administration, supervision, validation, visualization, writing—original draft, writing—review and editing. Michael Chen: methodology, formal analysis, validation, visualization, writing—review and editing. Sergey Yurasov: conceptualization, data curation, formal analysis, supervision, writing—review and editing. Steven Attia: conceptualization, formal analysis, writing—review and editing.

## Supporting information

Supplementary Material

## Data Availability

Data sharing is not applicable to this article as no new data were created or analyzed in this study.

## References

[1] Tuna M , Ju Z , Amos CI , Mills GB . Soft tissue sarcoma subtypes exhibit distinct patterns of acquired uniparental disomy. BMC Med Genomics. 2012;5:60. 23217126 10.1186/1755-8794-5-60 PMC3541987

[2] Ducimetière F , Lurkin A , Ranchère‐Vince D , et al. Incidence of sarcoma histotypes and molecular subtypes in a prospective epidemiological study with central pathology review and molecular testing. PLoS ONE. 2011;6(8):e20294. 21826194 10.1371/journal.pone.0020294 PMC3149593

[3] Stacchiotti S , Van Tine BA . Synovial sarcoma: current concepts and future perspectives. J Clin Oncol. 2018;36(2):180‐187. 29220290 10.1200/JCO.2017.75.1941

[4] Italiano A , Le Cesne A , Blay JY , et al. Patterns of care and outcome of patients with metastatic soft‐tissue sarcoma according to histological subtype and treatment setting: the METASTAR Study. J Clin Oncol. 2016;34(suppl 15):11014.

[5] Vlenterie M , Litiere S , Rizzo E , et al. Outcome of chemotherapy in advanced synovial sarcoma patients: review of 15 clinical trials from the European Organisation for Research and Treatment of Cancer Soft Tissue and Bone Sarcoma Group; setting a new landmark for studies in this entity. Eur J Cancer. 2016;58:62‐72. 26968015 10.1016/j.ejca.2016.02.002

[6] Riedel R , Jones R , Italiano A , et al. Systemic anti‐cancer therapy in synovial sarcoma: a systematic review. Cancers (Basel). 2018;10(11):E417. 30388821 10.3390/cancers10110417 PMC6267101

[7] Hoffman A , Ghadimi MPH , Demicco EG , et al. Localized and metastatic myxoid/round cell liposarcoma: clinical and molecular observations. Cancer. 2013;119(10):1868‐1877. 23401071 10.1002/cncr.27847

[8] Riedel R . Systemic therapy for advanced soft tissue sarcomas: highlighting novel therapies and treatment approaches. Cancer. 2012;118(6):1474‐1485. 21837668 10.1002/cncr.26415 PMC3412982

[9] Van Glabbeke M , van Oosterom AT , Oosterhuis JW , et al. Prognostic factors for the outcome of chemotherapy in advanced soft tissue sarcoma: an analysis of 2,185 patients treated with anthracycline‐containing first‐line regimens–a European Organization for Research and Treatment of Cancer Soft Tissue and Bone Sarcoma Group Study. J Clin Oncol. 1999;17(1):150‐157. 10458228 10.1200/JCO.1999.17.1.150

[10] Spurrell EL , Fisher C , Thomas JM , Judson IR . Prognostic factors in advanced synovial sarcoma: an analysis of 104 patients treated at the Royal Marsden Hospital. Ann Oncol. 2005;16(3):437‐444. 15653701 10.1093/annonc/mdi082

[11] Tap WD , Papai Z , Van Tine BA , et al. Doxorubicin plus evofosfamide versus doxorubicin alone in locally advanced, unresectable or metastatic soft‐tissue sarcoma (TH CR‐406/SARC021): an international, multicentre, open‐label, randomised phase 3 trial. Lancet Oncol. 2017;18(8):1089‐1103. 28651927 10.1016/S1470-2045(17)30381-9 PMC7771354

[12] Demetri GD , von Mehren M , Jones RL , et al. Efficacy and safety of trabectedin or dacarbazine for metastatic liposarcoma or leiomyosarcoma after failure of conventional chemotherapy: results of a phase III randomized multicenter trial. J Clin Oncol. 2016;34(8):786‐793. 26371143 10.1200/JCO.2015.62.4734 PMC5070559

[13] Schöffski P , Chawla S , Maki RG , et al. Eribulin versus dacarbazine in previously treated patients with advanced liposarcoma or leiomyosarcoma: a randomised, open‐label, multicenter, phase 3 trial. Lancet. 2016;387(10028):1629‐1637. 26874885 10.1016/S0140-6736(15)01283-0

[14] Banito A , Li X , Laporte AN , et al. The SS18‐SSX hijacks KDM2B‐PRC1.1 to drive synovial sarcoma. Cancer Cell. 2018;33(3):527‐541.e8. 29502955 10.1016/j.ccell.2018.01.018 PMC5881394

[15] McBride MJ , Pulice JL , Beird HC , et al. The SS18‐SSX fusion oncoprotein hijacks BAF complex targeting and function to drive synovial sarcoma. Cancer Cell. 2018;33(6):1128‐1141.e7. 29861296 10.1016/j.ccell.2018.05.002 PMC6791822

[16] Yu JSE , Colborne S , Hughes CS , Morin GB , Nielsen TO . The FUS‐DDIT3 interactome in myxoid liposarcoma. Neoplasia. 2019;21(8):740‐751. 31220736 10.1016/j.neo.2019.05.004 PMC6584455

[17] Somaiah N , Block MS , Kim JW , et al. First in‐class, first in‐human study evaluating LV305, a dendritic‐cell tropic lentiviral vector in sarcoma and other solid tumors expressing NY‐ESO‐1. Clin Cancer Res. 2019;25(19):5808‐5817. 31227504 10.1158/1078-0432.CCR-19-1025

[18] Zhang S , Kohli K , Black RG , et al. Systemic interferon‐γ increases MHC class I expression and T‐cell infiltration in cold tumors: results of a phase 0 clinical trial. Cancer Immunol Res. 2019;7(8):1237‐1243. 31171504 10.1158/2326-6066.CIR-18-0940 PMC6677581

[19] Pollack SM , He Q , Yearley JH , et al. T‐cell infiltration and clonality correlate with programmed cell death protein 1 and programmed death‐ligand 1 expression in patients with soft tissue sarcomas. Cancer. 2017;123(17):3291‐3304. 28463396 10.1002/cncr.30726 PMC5568958

[20] Dürr HR , Rauh J , Baur‐Melnyk A , et al. Myxoid liposarcoma: local relapse and metastatic pattern in 43 patients. BMC Cancer. 2018;18(1):304. 29558901 10.1186/s12885-018-4226-8 PMC5859402

[21] Muratori F , Bettini L , Frenos F , et al. Myxoid liposarcoma: Prognostic factors and metastatic pattern in a series of 148 patients treated at a single institution. Int J Surg Oncol. 2018;2018:8928706. 29977616 10.1155/2018/8928706 PMC6011058

[22] Van der Graaf WTA , Blay J‐Y , Chawla SP , et al. Pazopanib for metastatic soft tissue sarcoma (PALETTE): a randomised, double‐blind, placebo‐controlled phase 3 trial. Lancet. 2012;379(9829):1879‐1886. 22595799 10.1016/S0140-6736(12)60651-5

[23] Nakamura T , Matsumine A , Kuwai A , et al. The clinical outcomes of pazopanib treatment in Japanese patients with relapsed soft tissue sarcoma: a Japanese Musculoskeletal Oncology Group (JMOG) study. Cancer. 2016;122(9):1408‐1416. 26970174 10.1002/cncr.29961 PMC5069581

[24] Mir O , Brodowicz T , Italiano A , et al. Safety and efficacy of regorafenib in patients with advanced soft tissue sarcoma (REGOSARC): a randomised double‐blind, placebo‐controlled phase 2 trial. Lancet Oncol. 2016;17(12):1732‐1742. 27751846 10.1016/S1470-2045(16)30507-1

[25] Savina M , Le Cesne A , Blay J‐Y , et al. Patterns of care and outcomes of patients with METAstatic soft tissue SARComa in a real‐life setting: the METASARC observational study. BMC Med. 2017;15(1):78. 28391775 10.1186/s12916-017-0831-7 PMC5385590

[26] Singhi ER , Moore DC , Muslimani A . Metastatic soft tissue sarcomas: a review of treatment and new pharmacotherapies. P.T. 2018;43(7):410‐429. 30013298 PMC6027857

[27] Kawai A , Araki N , Sugiura H , et al. Trabectedin monotherapy after standard chemotherapy versus best supportive care in patients with advanced, translocation‐related sarcomas: a randomised open label phase 2 study. Lancet Oncol. 2015;16(4):406‐416. 25795406 10.1016/S1470-2045(15)70098-7

[28] Judson I , Verweij J , Gelderblom H , et al. Doxorubicin alone versus intensified doxorubicin plus ifosfamide for first‐line treatment of advanced or metastatic soft‐tissue sarcoma: a randomised controlled phase 3 trial. Lancet Oncol. 2014;15(4):415‐423. 24618336 10.1016/S1470-2045(14)70063-4

[29] Levine MN , Julian JA . Registries that show efficacy: good but not good enough. J Clin Oncol. 2008;26(33):5316‐5319. 18854560 10.1200/JCO.2008.18.3996

[30] Gliklich RE , Dreyer NA , Leavy MB (eds). Registries for evaluating patient outcomes: A user's guide (3rd ed.). Rockville, MD: Agency for Healthcare Research and Quality (US); 2014. 24945055

