# Supplementary material for: Clinical outcomes of patients with advanced synovial sarcoma or myxoid/round cell liposarcoma treated at major cancer centers in the United States
Source: Cancer Med. 2020 May 6;9(13):4593–602. doi: 10.1002/cam4.3039 (PMC7333839; doi:10.1002/cam4.3039)
Supplement: Supplementary file 1 — Supplementary Material [file CAM4-9-4593-s001.docx]

**Supporting Information Figure 1: Synovial Sarcoma: Progression-Free Survival and Prognostic Variables from First-Line Treatment**

**Supporting Information Figure 2. Myxoid/Round Cell Liposarcoma. Progression-Free Survival and Prognostic Variables from First-Line Treatment**

Supporting Information Figure 3. Overall Survival Outcomes by Each Therapy in Second-Line Treatment

**a) Synovial sarcoma: Overall survival by each second-line therapy with 20 or more patients**


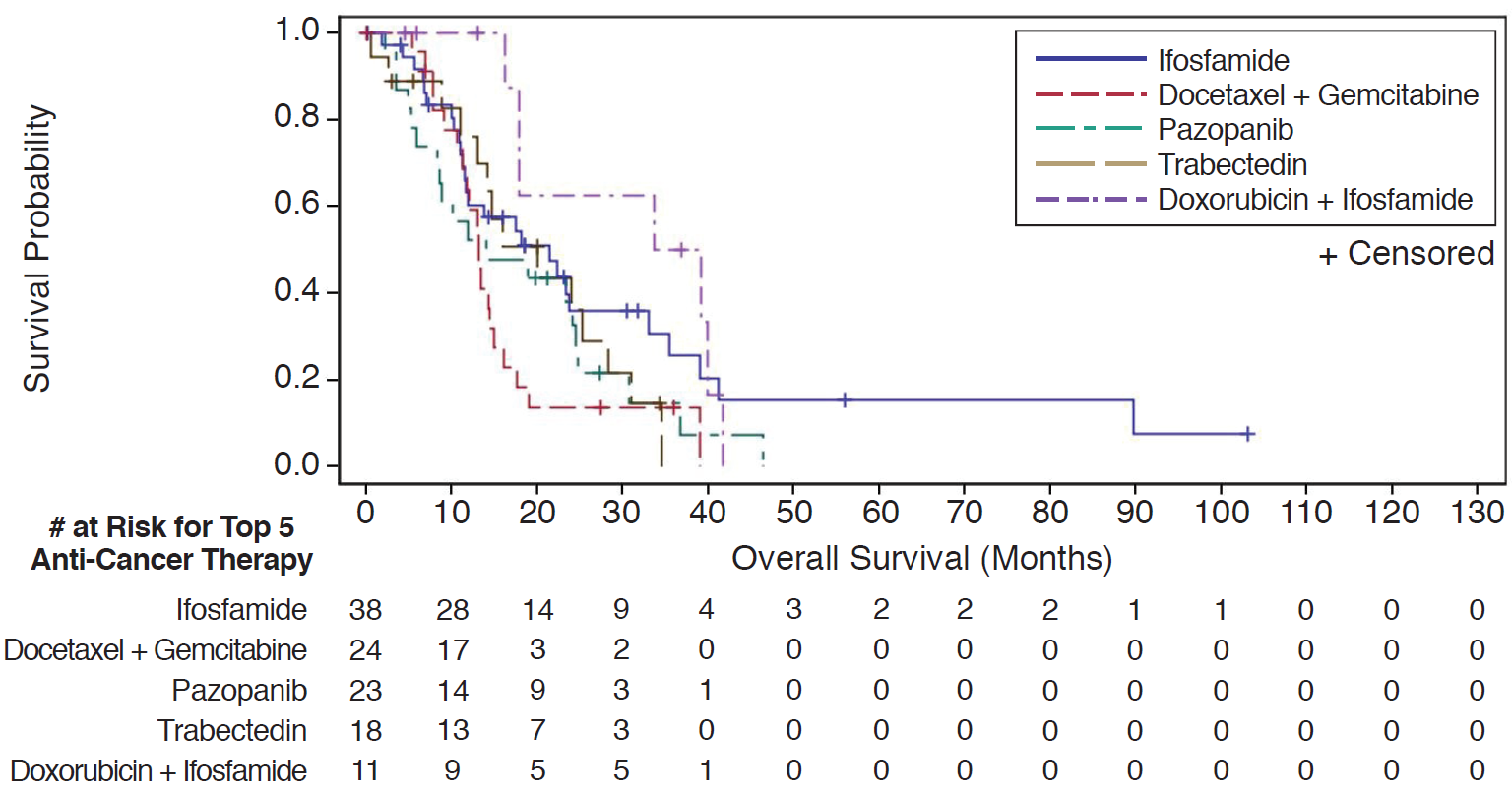


**b) Myxoid/round cell liposarcoma: Overall survival by each second-line therapy with 20 or more patients**


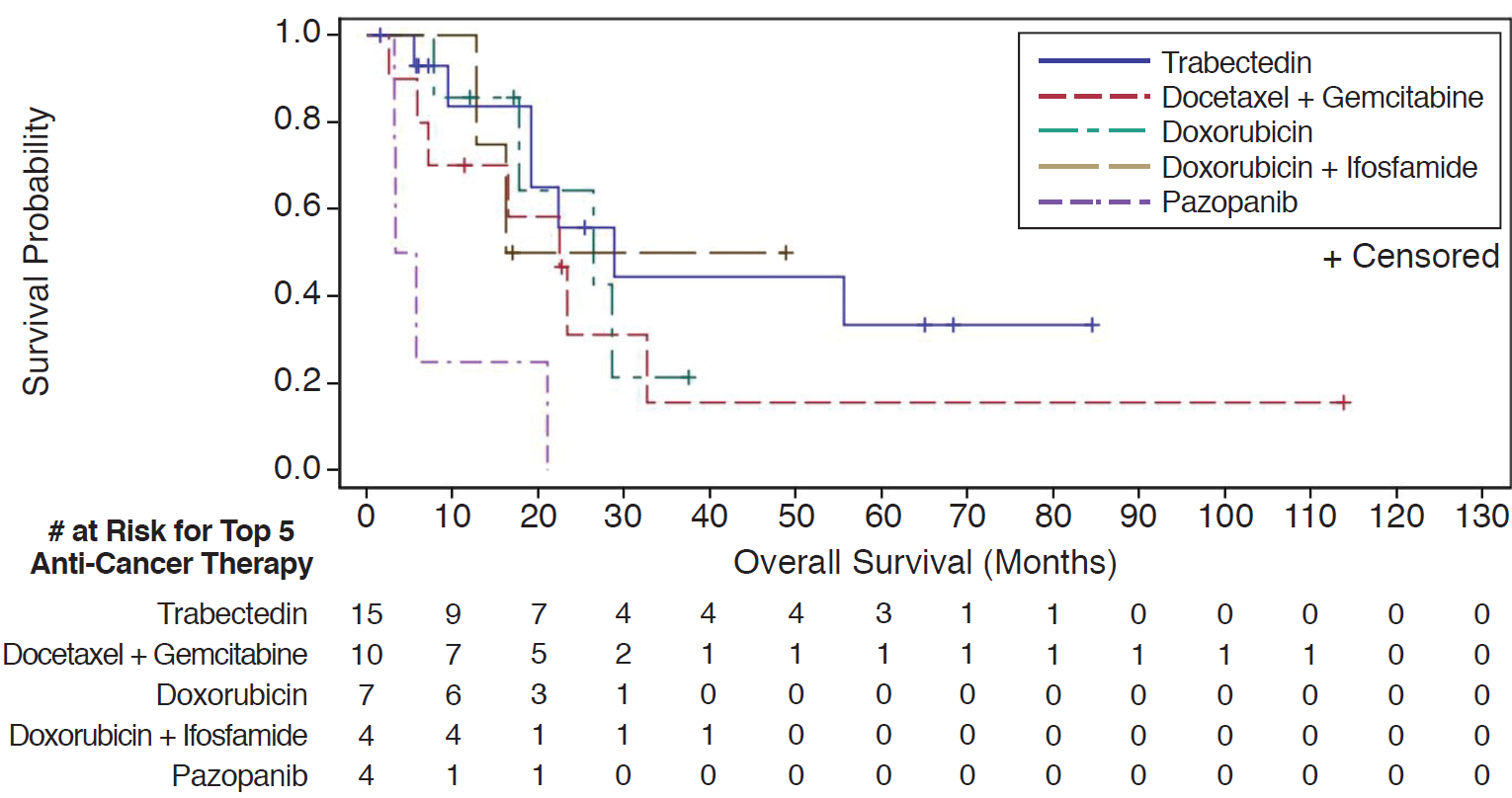


Supporting Information Figure 4. Overall Survival from First-Line Therapy by Site

**a) Synovial sarcoma**


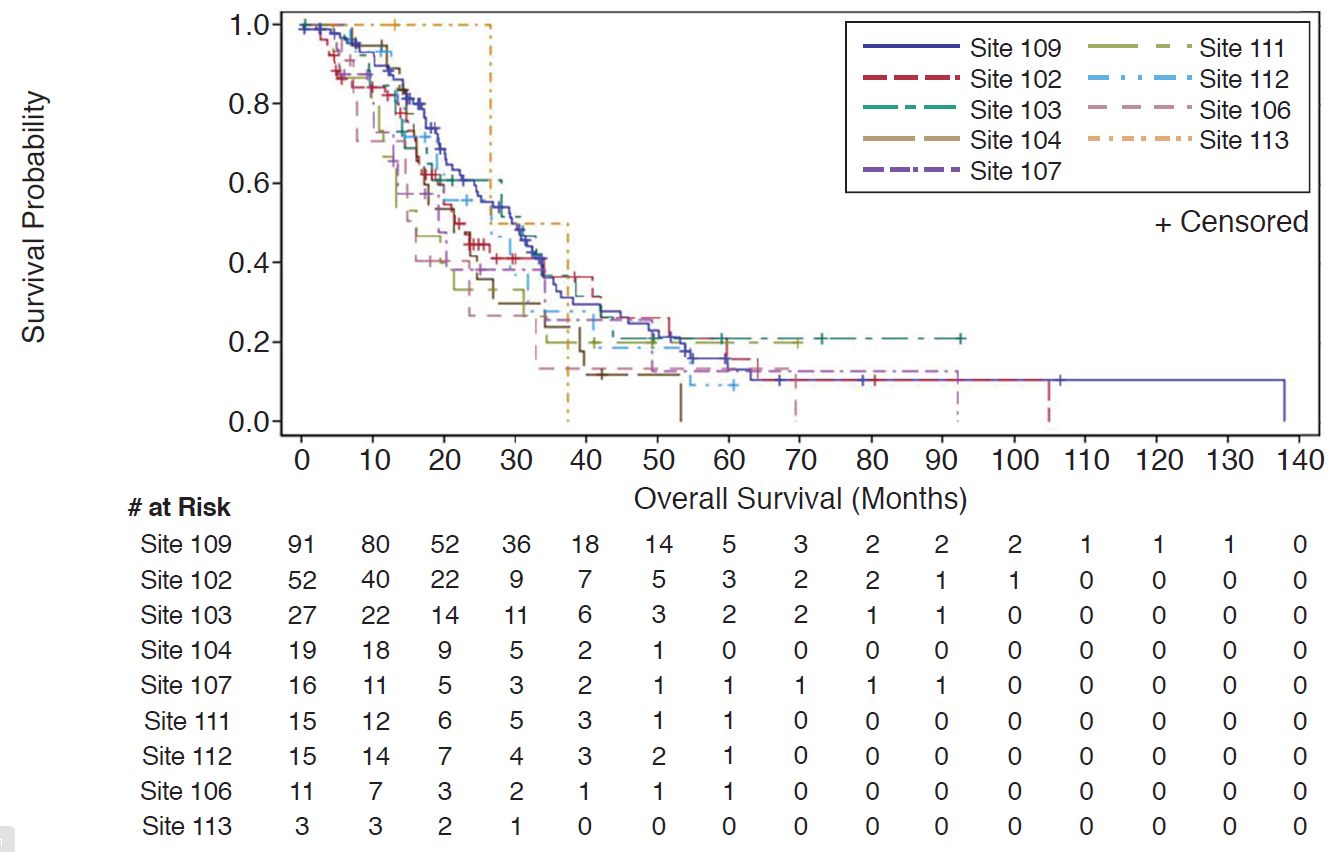


**b) Myxoid/round cell liposarcoma**


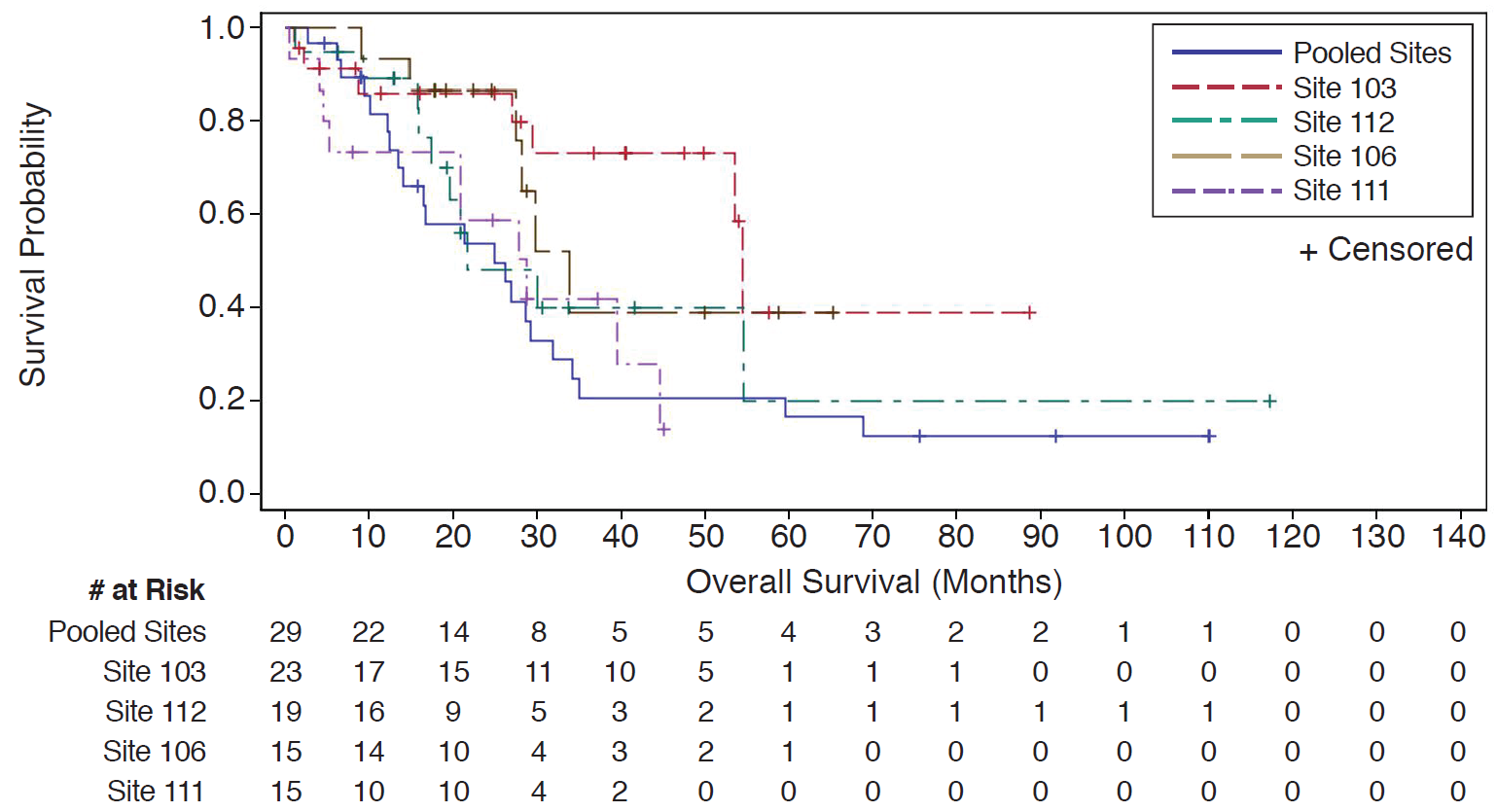


Supporting Information Figure 5. Progression-Free Survival from First-Line Therapy by Site

**a) Synovial sarcoma**


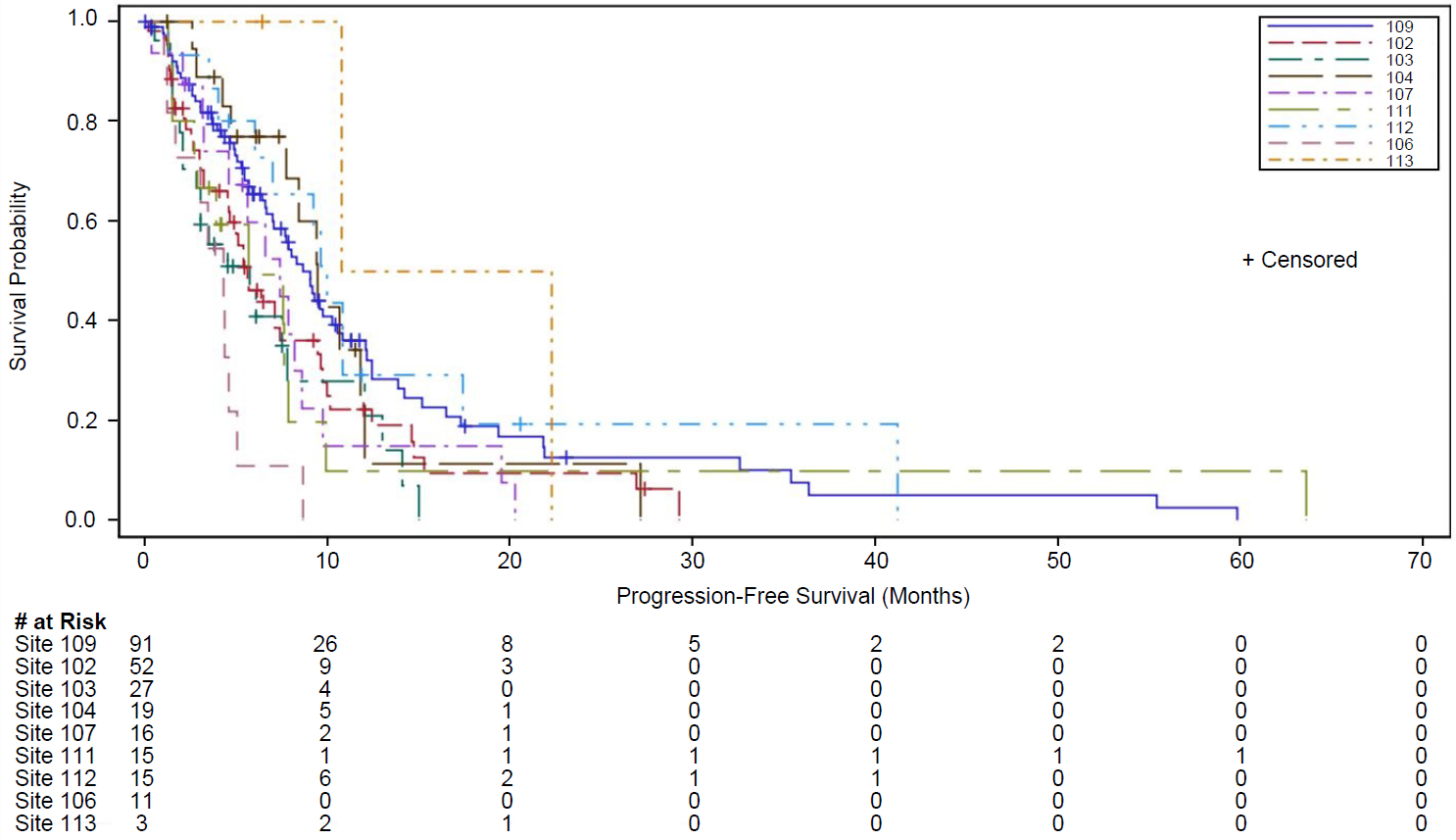


**b) Myxoid/round cell liposarcoma**


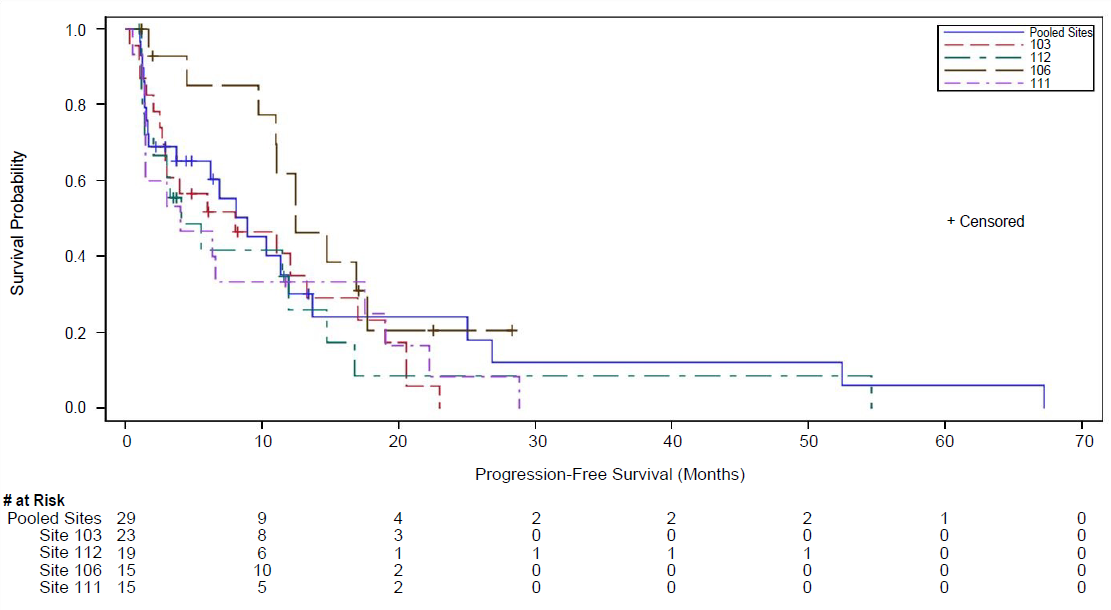


Note: Pooled sites includes Site 102 (10 patients), Site 107 (9 patients), Site 104 (8 patients), and Site 113 (2 patients).

Supporting Information Table 1. Patient Baseline Characteristics

| **Patient Characteristics and Cancer History** | **Synovial Sarcoma (N = 249)** | **Myxoid/Round Cell Liposarcoma (N = 101)** |
| --- | --- | --- |
|  | **n (%)** | **n (%)** |
| **Age (median)** | 40.0 | 50.0 |
| **Sex** |  |  |
| Male | 145 (58.2%) | 68 (67.3%) |
| Female | 104 (41.8%) | 33 (32.7%) |
| **Race** |  |  |
| American Indian or Alaska Native | 3 (1.2%) | 0 |
| Asian | 13 (5.2%) | 2 (2.0%) |
| Black or African American | 10 (4.0%) | 3 (3.0%) |
| Middle Eastern or Pacific Islander | 5 (2.0%) | 0 |
| White | 187 (75.1%) | 79 (78.2%) |
| Other | 9 (3.6%) | 0 |
| Unknown | 22 (8.8%) | 17 (16.8%) |
| **Stage at Diagnosis** |  |  |
| Stage I | 5 (2.0%) | 2 (2.0%) |
| Stage II | 36 (14.5%) | 22 (21.8%) |
| Stage III | 97 (39.0%) | 30 (29.7%) |
| Stage IV | 61 (24.5%) | 18 (17.8%) |
| Unknown | 50 (20.1%) | 29 (28.7%) |
| **Disease Status** |  |  |
| Locally Advanced | 37 (14.9%) | 9 (8.9%) |
| Metastatic | 212 (85.1%) | 92 (91.1%) |
| **Histologic Grade of Tumor** | (n = 247) | (n = 101) |
| Grade 1 | 1 (0.4%) | 24 (23.8%) |
| Grade 2 | 25 (10.0%) | 18 (17.8%) |
| Grade 3 | 150 (60.2%) | 38 (37.6%) |
| Unknown | 71 (20.5%) | 21 (20.8%) |

**Supporting Information Table 2. Baseline Laboratory Values**

| **Baseline Laboratory Values** | **Synovial Sarcoma**  **(N = 249)** | | **Myxoid/Round Cell Liposarcoma**  **(N = 101)** | |
| --- | --- | --- | --- | --- |
|  | **n** | **Median** | **n** | **Median** |
| Baseline Hemoglobin (g/L)^1^ | 113 | 130.0 | 39 | 137.0 |
| Baseline Platelets (10^9^/L)^1^ | 113 | 0.309 | 39 | 0.266 |
| Baseline Neutrophils (10^9^/L)^1^ | 97 | 0.005 | 35 | 0.004 |
| Baseline Lymphocytes (10^9^/L)^1^ | 87 | 0.002 | 34 | 0.002 |
| Baseline Neutrophil to Lymphocyte Ratio^2^ | 86 | 3.148 | 33 | 3.179 |
| Baseline Albumin (g/L)^1^ | 97 | 42.0 | 32 | 40.0 |

^1^ Baseline is defined as the last non-missing measurement at the start of first line anti-cancer therapy.

^2^ Neutrophil to Lymphocyte Ratio = Baseline Neutrophil/Baseline Lymphocyte when both are not missing.

**Supporting Information Table 3. Tumor Characteristics**

| **Tumor Characteristics** | **Synovial Sarcoma**  **(N = 249)** | **Myxoid/Round Cell Liposarcoma**  **(N = 101)** |
| --- | --- | --- |
|  | **n (%)** | **n (%)** |
| **Primary Tumor Size** | **(n = 185)** | **(n = 80)** |
| < 5 cm | 30 (12.0%) | 7 (6.9%) |
| ≥ 5 cm | 140 (56.2%) | 69 (68.3%) |
| Unknown | 15 (6.0%) | 4 (4.0%) |
| **Organ Site** | **(n = 249)** | **(n = 101)** |
| Soft Tissue | 219 (88.0%) | 97 (96.0%) |
| Lung | 207 (83.1%) | 45 (44.6%) |
| Bone | 42 (16.9%) | 28 (27.7%) |
| Lymph | 26 (10.4%) | 18 (17.8%) |
| Liver | 17 (6.8%) | 11 (10.9%) |
| Retroperitoneum | 17 (6.8%) | 23 (22.8%) |
| Pleura | 13 (5.2%) | 1 (1.0%) |
| Central Nervous System | 7 (2.8%) | 2 (2.0%) |
| Adrenal Gland | 6 (2.4%) | 0 |
| Renal | 4 (1.6%) | 0 |
| Bladder | 0 | 1 (1.0%) |
| **Primary or Metastatic Lesion** | **(n = 249)** | **(n = 101)** |
| Primary | 248 (99.6%) | 101 (100.0%) |
| Metastatic | 230 (92.4%) | 96 (95.0%) |
| **Organ Site for Primary** | **(n = 248)** | **(n = 101)** |
| Soft Tissue | 192 (77.1%) | 88 (87.1%) |
| Lung | 29 (11.6%) | 2 (2.0%) |
| Bone | 10 (4.0%) | 5 (5.0%) |
| Retroperitoneum | 7 (2.8%) | 5 (5.0%) |
| Renal | 4 (1.6%) | 0 |
| Lymph | 2 (0.8%) | 1 (1.0%) |
| Pleura | 2 (0.8%) | 0 |
| Adrenal Gland | 1 (0.4%) | 0 |
| Liver | 1 (0.4%) | 0 |
| **Organ Site for Metastatic Lesion** | **(n = 230)** | **(n = 96)** |
| Lung | 185 (74.3%) | 43 (42.6%) |
| Soft Tissue | 79 (31.7%) | 74 (73.3%) |
| Bone | 35 (14.1%) | 26 (25.7%) |
| Lymph | 24 (9.6%) | 17 (16.8%) |
| Liver | 16 (6.4%) | 11 (10.9%) |
| Retroperitoneum | 13 (5.2%) | 21 (20.8%) |
| Pleura | 11 (4.4%) | 1 (1.0%) |
| Central Nervous System | 7 (2.8%) | 2 (2.0%) |
| Adrenal Gland | 5 (2.0%) | 0 |
| Renal | 1 (0.4%) | 0 |
| Bladder | 0 | 1 (1.0%) |

Supporting Information Table 4. Anticancer Therapy and Clinical Outcomes

| **Anticancer Treatment** | **SS (N = 249)** | **MRCL (N = 101)** |
| --- | --- | --- |
|  | **n (%)** | **n (%)** |
| **Number of Subjects in Lines of Therapy** |  |  |
| 1 | 249 (100%) | 101 (100%) |
| 2 | 186 (74.7%) | 73 (72.3%) |
| 3 | 136 (54.6%) | 49 (48.5%) |
| 4 | 86 (34.5%) | 28 (27.7%) |
| > 4 | 48 (19.3%) | 16 (15.8%) |
| **First Line** | **(n = 249)** | **(n = 101)** |
| Subjects with Metastectomies | 114 (45.8%) | 53 (52.5%) |
| Top 5 Anticancer Agents in First Line |  |  |
| Ifosfamide | 158 (63.5%) | 31 (30.7%) |
| Doxorubicin | 120 (48.2%) | 58 (57.4%) |
| Docetaxel | 26 (10.4%) | 15 (14.9%) |
| Gemcitabine | 26 (10.4%) | 13 (12.9%) |
| Vaccines | 16 (6.4%) | -- |
| Trabectedin | -- | 8 (7.9%) |
| Best Response in First Line |  |  |
| Complete Response (CR) | 12 (4.8%) | 4 (4.0%) |
| Partial Response (PR) | 79 (31.7%) | 16 (15.8%) |
| Stable Disease (SD) | 64 (25.7%) | 38 (37.6%) |
| Progressive Disease (PD) | 67 (26.9%) | 33 (32.7%) |
| Unknown | 27 (10.8%) | 10 (9.9%) |
| Disease Stabilization Rate in First Line (CR + PR + SD) | 155 (62.2%) | 58 (57.4%) |
| **Second Line** | **(n = 186)** | **(n = 73)** |
| Subjects with Metastectomies | 24 (9.6%) | 11 (10.9%) |
| Top 5 Anticancer Agents in Second Line |  |  |
| Ifosfamide | 57 (22.9%) | 8 (7.9%) |
| Gemcitabine | 34 (13.7%) | 12 (11.9%) |
| Docetaxel | 28 (11.2%) | 11 (10.9%) |
| Pazopanib | 26 (10.4%) | -- |
| Doxorubicin | 24 (9.6%) | 13 (12.9%) |
| Trabectedin | -- | 15 (14.9%) |
| Best Response in Second Line |  |  |
| CR | 3 (1.2%) | 2 (2.0%) |
| PR | 33 (13.3%) | 8 (7.9%) |
| SD | 48 (19.3%) | 20 (19.8%) |
| PD | 67 (26.9%) | 36 (35.6%) |
| Unknown | 36 (14.5%) | 7 (6.9%) |
| Disease Stabilization Rate in Second Line (CR + PR + SD) | 84 (33.7%) | 30 (29.7%) |

**Supporting Information Table 5. Tumor Recurrence**

| **Pattern of Tumor Recurrence** | **Synovial**  **Sarcoma (N = 249)** | **Myxoid/Round Cell Liposarcoma (N = 101)** |
| --- | --- | --- |
|  | **n (%)** | **n (%)** |
| **First Line** | **(n = 249)** | **(n = 101)** |
| Subjects with New Metastases | 131 (52.6%) | 68 (67.3%) |
| Organ Site | (n = 131) | (n = 68) |
| Lung | 93 (37.3%) | 20 (19.8%) |
| Soft Tissue | 49 (19.7%) | 55 (54.5%) |
| Bone | 12 (4.8%) | 13 (12.9%) |
| Lymphatic System | 8 (3.2%) | 7 (6.9%) |
| Retroperitoneum | 8 (3.2%) | 8 (7.9%) |
| Liver | 6 (2.4%) | 4 (4.0%) |
| Adrenal Gland | 2 (0.8%) | 0 |
| Brain | 2 (0.8%) | 1 (1.0%) |
| Bowel | 0 | 1 (1.0%) |
| **Second Line** | **(n = 186)** | **(n = 73)** |
| Subjects with New Metastases | 84 (33.7%) | 36 (35.6%) |
| Organ Site | (n = 84) | (n = 36) |
| Lung | 51 (20.5%) | 7 (6.9%) |
| Soft Tissue | 37 (14.9%) | 20 (19.8%) |
| Brain | 7 (2.8%) | 0 |
| Liver | 6 (2.4%) | 6 (5.9%) |
| Bone | 5 (2.0%) | 7 (6.9%) |
| Lymphatic System | 4 (1.6%) | 3 (3.0%) |
| Retroperitoneum | 2 (0.8%) | 4 (4.0%) |

Supporting Information Table 6. Patient Disposition by Site

| **Site** | **Synovial Sarcoma (N = 249)** | **Myxoid/Round Cell Liposarcoma (N = 101)** |
| --- | --- | --- |
|  | **n (%)** | **n (%)** |
| Site 102 | 52 (20.9%) | 10 (9.9%) |
| Site 103 | 27 (10.8%) | 23 (22.8%) |
| Site 104 | 19 (7.6%) | 8 (7.9%) |
| Site 106 | 11 (4.4%) | 15 (14.9%) |
| Site 107 | 16 (6.4%) | 9 (8.9%) |
| Site 109 | 91 (36.5%) | 0 |
| Site 111 | 15 (6.0%) | 15 (14.9%) |
| Site 112 | 15 (6.0%) | 19 (18.8%) |
| Site 113 | 2 (1.2%) | 2 (2.0%) |
